# Assessment of San Antonio refugee health clinic telehealth response and refugees’ needs during the COVID-19 pandemic

**DOI:** 10.1016/j.jmh.2023.100190

**Published:** 2023-05-08

**Authors:** Salma Yazji, Pavan Mahima Chowdhry Ginjupalli, Zachary Harbin, Nurani Kester, Abhishek Roka

**Affiliations:** Center for Medical Humanities and Ethics, The University of Texas Health San Antonio, 7703 Floyd Curl Dr, San Antonio, TX 78229, United States

## Abstract

In March of 2020, Texas declared a statewide public health emergency in response to the rapidly spreading COVID-19 virus, forcing the shutdown of many critical operations across the state. The pandemic has had a massive impact on the refugee population worldwide, increasing displacement and limiting opportunities for resettlement, work, and aid. In an effort to evaluate and address the holistic needs of the San Antonio's vulnerable refugee community during the pandemic, the San Antonio Refugee Health Clinic (SARHC) created a COVID-19 response team that screened and triaged the population, collected data, and provided telemedicine and other urgent teleservices.

The SARHC clinic has served the mostly uninsured and underserved refugee population of San Antonio, Texas for over 10 years as a Student-Faculty Collaborative Practice (SFCP). With the collaboration of the Center for Refugee Services in San Antonio, the clinic utilizes the site of a local church on a weekly basis to serve refugees via teams of nursing, dental, and medical students and faculty.

At the height of the COVID-19 lockdown, teams of student and faculty volunteers conducted a cross-sectional study of patients’ needs by systematically calling and screening patients. Qualitative data on COVID-19 risk, mental wellness, financial needs, food security, dental needs, and medical needs was collected. Quantitative data on number of patients contacted, country of origin, interpreter use, insurance access, internet access, referrals, appointments, and prescriptions carried out was also collected and analyzed.

Of the patients called (*N* = 216), 57% (*n* = 123) were successfully reached and completed the survey. 61% (*n* = 75) required language interpreter services. Only 9% (*n* = 11) of individuals had health insurance. 46% (*n* = 52) expressed the need for telemedicine services, and 34% (*n* = 42) reported access to WiFi. 41% (*n* = 50) reported a medical concern, 18% (*n* = 22) reported dental concerns, 41% (*n* = 51) reported social needs, and 11% (*n* = 14) reported mental health concerns. 24% (*n* = 30) of patients requested medication refills.

Our snapshot of the San Antonio refugee community during the COVID-19 pandemic captures their social, mental, and physical struggles; the pandemic left many families without access to medications, health services, social services, job, and food security. The telemedicine campaign proved to be an effective method of assessing and addressing a variety of patient needs in a virtual setting. Of concern is the high rates of uninsured families and limited Internet access. These findings reveal important considerations for equitable healthcare delivery to vulnerable populations in the face of prolonged unforeseen events, like the COVID-19 pandemic.

## 1 Introduction

According to the State Department's Bureau of Population, Refugees, and Migration, Texas has received more refugees than any other state between 2010 and 2019, with nearly 57,000 total recorded arrivals (Refugee Processing Center 2016). Over 1000 refugees from over 20 countries resettle in Bexar County every year (Texas Department of State Health Services 2014), escaping persecution, violence, political strife, and human rights abuses to rebuild their livelihoods in San Antonio (Anon, Inpress). Many of these families face a period of significant adjustment and change that puts strain on their physical, mental, and emotional wellbeing, on top of the trauma they already left their homes with (Texas School Health Program, Inpress). A lot of this strain comes with current Texas guidelines, in which most refugees lose their Medicaid health insurance coverage after 8 months of resettlement (US Department of Health and Human Services, Inpress), rendering them without proper access to healthcare.

In 2011, the University of Texas Health Center at San Antonio (UTHSCSA) partnered with the Center for Refugee Services, a nonprofit organization that promotes the wellbeing, self-sufficiency, and community integration of resettled refugee families (3). Together they formed the San Antonio Refugee Health Clinic (SARHC), an interprofessional student-run clinic that addresses the health and social needs of the vulnerable refugee population (Anon, Inpressb). Every Wednesday in the St. Francis Episcopal Church (SFEC), the clinic opens its doors to dozens of refugees and their families seeking free health screenings, medical examinations, dental services, psychological evaluations, vaccinations, prescriptions, and referrals (Anon, Inpressb). According to a study conducted within SARHC, 86.6% of patients seen at the clinic do not have any form of insurance (Adel et al., 2019). Uncontrolled hypertension and diabetes, chronic pain, dental needs, and preventable illnesses make up a significant portion of the patients’ health concerns (Adel et al., 2019).

SARHC typically hosts an average of ten medical students, ten nursing students, five dental students, five attending physicians, and one dentist every week. The clinic utilizes many paid-by-the-hour in-house interpreters who bridge the cultural and language divide while fostering the relationship between the patient and entire healthcare team. The clinic consists of six examination rooms with basic medical equipment, along with an in-house pharmacy. Patients are seen on a walk-in, first-come first-served basis (Table 1).

**Table 1:** Medical chief complaints of refugee patients requesting telehealth appointments.

Chief Complaint	Number of Patients (%)
Musculoskeletal pain	20 (20.6)
Diabetes or HTN Follow up	14 (14.4)
Constitutional symptoms, headache	12 (12.4)
Lab Follow up / Other	12 (12.4)
GI complaint	9 (9.3)
General check-up	8 (8.2)
Obstetric or gynecology complaint	5 (5.2)
Upper respiratory symptoms	5 (5.2)
Vision problems	4 (4.1)
Dermatological complaint	3 (3.1)
Cardiovascular concern	3 (3.1)
Neurological complaint	2 (2.1)
Total	97 (100)

In March of 2020, Texas declared a statewide public health emergency in response to the rapidly spreading COVID-19 virus (Salinas, 2020). This sudden development resulted in the unannounced closure of the Student Run Free Clinics by UTHSCSA, including SARHC. Worldwide, the COVID-19 pandemic has had a massive impact on the refugee population, increasing displacement and limiting opportunities for resettlement, work, and aid (Ritchie, 2021). In response to the possible effects of the COVID-19 pandemic and resultant city closure on San Antonio's refugee community, SARHC created a COVID-19 response team consisting of medical, nursing, and dental students and faculty. The team designed and implemented a new, unprecedented plan focused on screening the population for its social, physical, and mental needs during the pandemic, while providing telemedicine and other teleservices for the first time in the clinic's history.

This cross-sectional study was undertaken to assess the burden of COVID-19 on the needs of the San Antonio refugee population. The results have been utilized to inform community partners and clinical team on how to best allocate resources for the population during this public health crisis. In the process, the team was also able to identify the countries of origin, spoken languages, common symptomatology, social histories, insurance status, labs ordered, prescribed medications, and the prevalence of certain chronic diseases among the most vulnerable members of the population. The resulting understanding of this unique community will allow us to better serve their needs moving forward while providing a foundation for planning and response strategies for similar clinics also facing unforeseen events in the future.

## 2 Material and methods

A comprehensive list of patients seen over the six months prior to clinic closure (September 2019 – March 2020) was compiled using a secure SharePoint spreadsheet. A screening questionnaire was developed with the clinic's physician staff and San Antonio Center for Refugee Services community partners to target needs of the patient refugee community during the COVID-19 pandemic. Student and faculty volunteers from the University of Texas Health Science Center were trained by clinic leaders via Zoom on how to administer the questionnaire using a newly established clinic Google Voice line and a third-party professional interpretation line for encounters in which a language interpreter was necessary. Each volunteer was assigned a set number of patients to call every week and patient responses were recorded in the shared secure spreadsheet. Volunteers were instructed to re-attempt contact throughout the week for patients not reached on first contact, recording on the spreadsheet every time a voice mail was left and/or phone call was attempted. Those who dealt with disconnected phone lines or incorrect phone numbers contacted CRS to cross-reference the patient's contact information with the center's most updated records. Any patients who screened positive for medical, dental, mental health, and/or social needs were forwarded weekly to the appropriate team for follow-up.

**Table:** 

**San Antonio Refugee Health Clinic Telehealth Screening Form**		
*COVID-19 Screening*		
Have you or anyone in your household been diagnosed with COVID-19?	**YES**	**NO**
In the past month, have you or anyone in your household had a fever, shortness of breath, and/or a cough?	**YES**	**NO**
In the past month, have you or anyone in your household traveled outside the city?	**YES**	**NO**
*Mental Health Screening*		
In the past month, have you:		
Been feeling down or sad most of the days?	**YES**	**NO**
Had too many thoughts or been distracted with too much thinking?	**YES**	**NO**
Been feeling emotionally numb (for example, feeling sad but cannot cry, or unable to have loving emotions)?	**YES**	**NO**
*Social Screening*		
Are you and/or your family struggling with:		
Paying bills?	**YES**	**NO**
Accessing food?	**YES**	**NO**
Employment?	**YES**	**NO**
Other basic needs?	*Elaborate.*	
*Medical Screening*		
Do you need medication refills?	*Medication, Dosage, Pharmacy information*	
Would you like to schedule a telehealth call with our team?	**YES**	**NO**
Medical, dental, and/or mental health concern?		
Reason(s) for appointment.		
WiFi access?	**YES**	**NO**

The prescreening information required the UT Health volunteers have access to the clinic electronic medical records, RedCap, to record basic patient information along with primary language, need of a translator for communication, and date of last visit.

Patients were screened for COVID-19 exposure or history with three Y/N questions which focused on symptoms, diagnosis, or exposure within the family in the past month. Patients who screened positive for any of the questions were referred to the clinic's medical director for further evaluation to determine whether the patient needed to receive a COVID-19 test from a local site and/or quarantine. Patients were informed to report to an urgent clinic or emergency room if severe symptoms occurred.

Patients were screened for mental health needs with three Y/N questions taken from the Refugee Health Screener-15 questionnaire, an empirically developed tool that screens refugees for distressing mental health symptoms. Patients who screened positive for any of the questions were referred to the clinic's psychiatric faculty. The clinic's psychiatric faculty conducted follow-up appointments via telehealth calls on the first Wednesday of every month with exceptions being made for patients that showed particular need for urgent psychiatric care.

Patients were screened for social needs (regarded as especially urgent during the pandemic) with three Y/N questions and one “elaborate” question that assess financial, housing, food access, employment, and other needs. Patients who screened positive for social needs were referred to San Antonio Center for Refugee Services with a detailed description regarding the patient and their social need given.

Patients were additionally screened for medication refill need. Established patients who reported the need for a medication refill were initially scheduled for a telehealth appointment with physician faculty for a checkup unless the refill was deemed to be urgent. Medication information was collected and called into the patient's requested pharmacy for pickup after 24 h.

Patients were screened for dental needs with an open-ended question. If the patient reported any dental pain or concerns, the screener administered a specialized questionnaire developed by the clinic's dental faculty to determine the appropriate resources for the patient. The response to oral health needs of patients were directed by the dental team, headed by the clinic's dental director who then reached out and coordinated with the patients to provide dental care.

Patients were screened for medical needs with an open-ended question. If the patient said they had a medical concern or wanted to speak to a physician, a chief complaint and brief history was obtained and documented within the spreadsheet. A telehealth appointment was scheduled for these patients on the weekly clinic night. Clinic nights typically consisted of three providers along with the three medical and four nursing student leaders. The visit would be conducted over the phone and appropriate referrals, labs, and prescriptions would be given to the patient. Should the patient request an interpreter, a three-way call was made using the InterpreTalk phone service. Any patient requiring a prescription would be able to pick up their medication at their preferred pharmacy the next day after the physician called it in. Lab forms were filled out by the medical team virtually and forwarded to the outpatient lab at University Hospital, where patients would get bloodwork done free of charge. Patients were then scheduled for a follow-up with the medical team the following week to discuss results. Throughout this process, patients had access to the medical team through the Google Voice line through either text message or phone call as needed.

## 3 Results

Between the dates of June 10th, 2020 and August 25th , 2020, fifteen volunteers attempted to contact 216 patients whose last date of visit to the clinic ranged from October 2019 to March 2020. There was a 58% success rate, with 123 out of 216 patients being successfully reached. Notes left for unreachable patients included disconnected phone lines, full voice mail boxes, and wrong phone numbers. The patients who were not reached the first time were called at least twice and a voice mail was left in their primary language. 58% of patients who were successfully reached were contacted on the first attempt (*n* = 71). 42% of patients (*n* = 52) reached were reached in subsequent calls.

Of the 123 patients reached, the survey was fully completed by 95 patients, with many of the remaining patients still responding to a few key questions. Reasons for the survey not being completed included patients no longer living in San Antonio, patients having access to another medical provider, or lack of time to complete the survey.

Of the 95 respondents, 22 originated from Afghanistan, 16 from Burma/Myanmar, 14 from Iraq, 8 from Iran, 7 from Nepal, 6 from Malaysia, and less than 5 from each of the following countries: India, Cameroon, Sudan, Lebanon, Eritrea, Turkey, Mexico, Congo, Syria, Jordan, Sri Lanka, Ethiopia, Ivory Coast, and Nigeria.

**Fig. 1:**
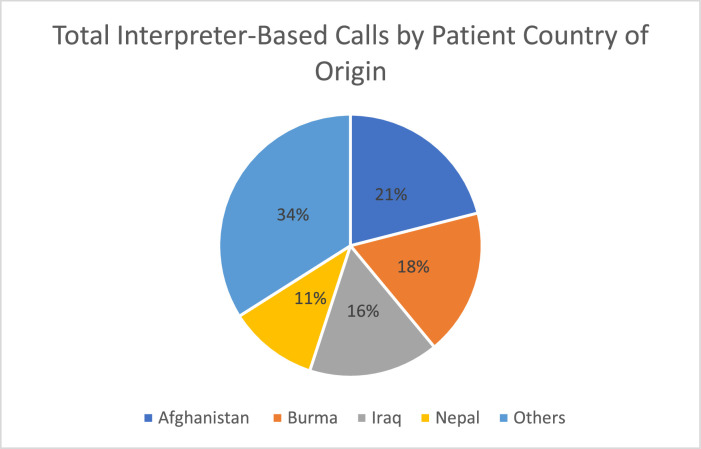
Overall interpreter service use by refugee patient country of origin.

**Fig. 2:**
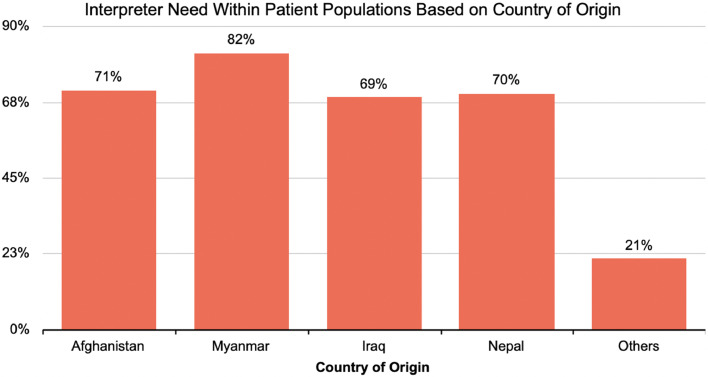
Proportion of refugee patients who utilized interpreter services by country of origin.

75 individuals (61%) required language interpreter services. InterpreTalk, a professional medical translator phone line, or multilingual volunteers were utilized to contact or leave voicemails for primarily Pashto or Dari speakers from Afghanistan, Rohingya speakers from Burma/Myanmar, Arabic speakers from Iraq, and Nepali speakers from Nepal. Interpreter services were overall utilized the most by patients from Afghanistan (Fig. 1) but when viewed by proportion within each patient population, patients from Burma/Myanmar requested interpreter services most often (82%) (Fig. 2).

In total, five individuals (4%) reported that in the past month, they or someone in their household had a fever, shortness of breath, and/or a cough. Three individuals reported recent travel. Those who screened positive for any of the COVID-19 screening questions were given information on precautions to take and triaged by the clinic's medical director.

**Fig. 3:**
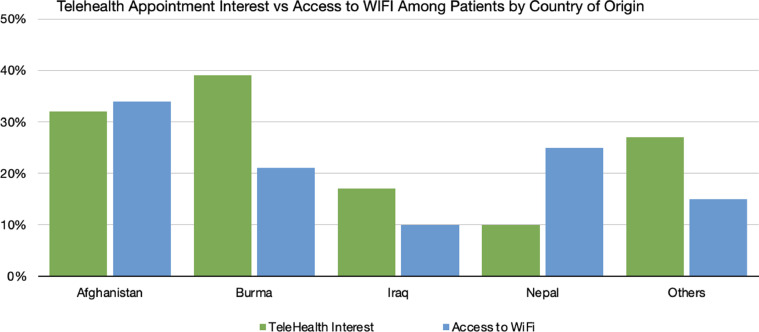
Proportion of refugee patients by country of origin with telehealth interest versus patients’ access to WIFI.

Only 11 individuals (9%) interviewed reported having health insurance. Although 56 individuals (46%) reported an interest in telehealth, of those, only 42 individuals (75%) reported access to WIFI, and 30 individuals (54%) had access to a webcam. Access to WIFI was a significant barrier particularly for Rohingya patients (Fig. 3).

**Fig. 4:**
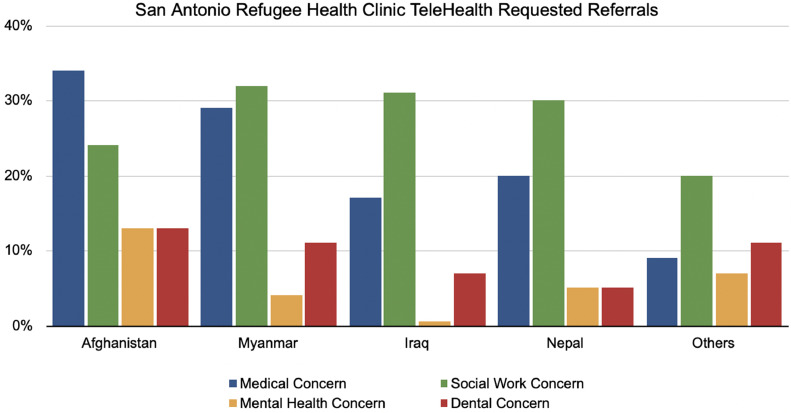
Proportion of refugee patients by country of origin with medical concern, social work concern, mental health concern, and/or dental concern.

Of the 123 individuals reached, 50 individuals (41%) reported a medical concern, 22 (18%) reported a dental concern, 51 (41%) reported a concern related to social services and 15 (12%) reported a mental health concern or wanted to speak with a psychiatrist (Fig. 4).

**Fig. 5:**
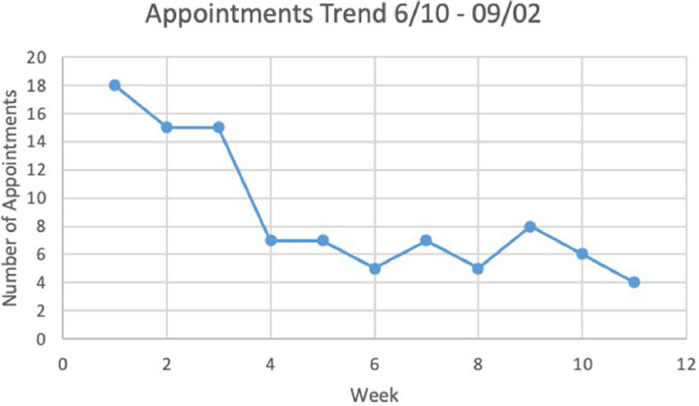
Number of medical appointments booked per week over 11 weeks.

When breaking down the social concerns among patients who responded, 30% (*n* = 29) of patients reported that they were struggling to pay their bills, 24% (*n* = 24) reported that they were struggling with access to employment, and 11% (*n* = 10) reported that they were struggling with access to food. Regarding the mental health screening component of the survey, 9% (*n* = 9) of patients reported emotional numbness, 13% (*n* = 13) reported anxiety, and 15% (*n* = 15) reported feelings of depression in the past month. Everyone who screened positive for a social or mental health concern followed up with a referral to Center for Refugee Services and psychiatric services through the clinic (Fig. 5).

Psychiatric care appointments and oral healthcare appointments for patients with those specific needs were scheduled and headed by designated Wellness Night Coordinators and Dental Student Leaders. 22 patients were triaged and referred to dental care through the telehealth effort and in addition Dental Student Leaders received and triaged another 38 patients through referrals from community partners. In total, 22 individuals received urgent dental care services through a multi-system, multi-provider approach in July, while others were referred out to community dental clinics.

30% (*n* = 37) of patients screened required medication refills. Most requested medications were antihypertensives (*n* = 10, 27%), followed by antidiabetic drugs (*n* = 8, 22%), antihistamines (*n* = 6, 16%), non-steroidal anti-inflammatory drugs (NSAIDs) (*n* = 5, 14%), lipid lowering agents (*n* = 5, 14%), proton pump inhibitors (*n* = 4, 11%), antibiotics and antifungals (*n* = 2, 5%), anticonvulsants (*n* = 1, 3%), and hormone replacement therapy (*n* = 1, 3%).

Of the 171 responses based on previous Redcap data, 30% of patients (*n* = 50) were expecting a follow up appointment at the clinic and 14% (*n* = 24) were expecting lab results before the pandemic caused the clinic to shut its doors. Once telemedicine appointments where underway, there was an average of 9 appointments per week, with the number of appointments decreasing per week. A total of 97 telehealth appointments were given. The most common chief complaint was musculoskeletal pain (*n* = 20, 20.6%), followed by a diabetes or hypertension follow-up (*n* = 14, 14.4%), then constitutional symptoms (*n* = 12, 12.4%) and lab follow-up (*n* = 12, 12.4%).

## 4 Discussion

With the onset of the clinic closure at the height of the COVID-19 pandemic, the SARHC staff and patients were faced with an obstacle that was unprecedented in its ten years of service. The clinic previously depended solely on face-to-face, in-person services without the establishment of a clinic phone number for patients to reach the clinic outside of hours. The clinic team was tasked with figuring out how to systematically contact its patients, majority of whom are non-native English speakers living without reliable internet access, and how to deliver and target telehealth and tele-social services for their unique needs during the city's lockdown mandate. A significant component of this effort depended on the clinic's collaboration with available community partners and resources, including the Center for Refugee Services, the Christian Medical & Dental Associations, and the Wellness Night psychiatric team. The clinic's COVID-19 response team established open communication and weekly referral systems with these groups that allowed for secure follow-up with patients in need of specialized care.

*Doximity*, a telemedicine platform, was first considered as a potential means to conduct medical appointments. The platform has capacity to host a 3-way secure video-call between providers, patients, and clinic translators. However, during the screening calls, it was found that a significant number of patients did not have access to internet or webcams. It was also found that a considerable number of individuals needed urgent care in the first week of screening calls, as the clinic had been closed for 2 months at that point, so a decision was made to move appointments to a phone call format in order to expand access to care. Over time, *Zoom* was incorporated into clinic nights for the medical team which allowed medical students to scribe the patient encounter in real time and reduce work for providers. *Zoom* is deemed a HIPAA compliant platform and was thus considered secure. The virtual/telehealth format of the medical visits proved to be one of the biggest limitations in this endeavor. Although the clinic had the ability to send patients for labs and prescribe medications, without video capabilities or Wireless Stethoscopes/ECG machines, there was no way to conduct physical exams or have vitals on hand in real time.

One of the biggest benefits to come out of the initiative proved to be the establishment of the clinic Google Voice phone line. The phone line became the central line of communication with patients and streamlined the process of clinic volunteers contacting patients. Once a patient had our phone number, they were able to contact the clinic at any time, with most calls or texts being answered immediately or within 24 h. This was especially useful for patients who lacked reliable transportation or who had other obligations such as work during clinic hours. The phone line has remained an integral part of the clinic even since its reopening.

One challenge of the phone line was communicating with patients who spoke languages other than those of the clinic team members. As with any patient who speaks English as a second language, it is preferable to see the patient face to face with a trained translator present. We feared that the virtual mode of communication would make effective communication even more challenging. The utilization of *InterpreTalk*, a professional medical interpretation phone line capable of 3-way calls, and Google Translate for text conversations proved to be largely effective for setting up appointments, corresponding for labs, and addressing social needs of patients. We hope to utilize the clinic phone line for general automation of clinic services such as upcoming vaccination clinics or health initiatives for patients who opt-in for this function. It would also be helpful to develop a streamlined process to communicate with patients about lab appointments, prescription pick-ups, and appointment reminders or follow-ups.

Patients from our clinic faced a significant set of challenges during the pandemic as evidenced by the screening data collected. The most reported social struggle was with paying bills, followed by employment, and then food security. The most reported “other” social issue was access to housing, as many could not meet their rental payments and feared eviction. Most patients that screened positive for a social concern reported that as a result of the city shutdown, they were either laid off from their jobs or their hours were cut short. Most come from at least four-person households living paycheck-to-paycheck. These stressors in turned amplified feelings of anxiety and depression, as shown by the positive mental health screenings.

A sizable number of patients reported recent-onset mental health concerns involving stress, anxiety, and depressive symptoms. The reported results may be under-representative of the population's true mental health burden due to both the patients’ cultural stigma of discussing these topics or asking for help, and the 3-way communication system causing topics to be lost in translation. The economic and social instability experienced by individual families during the pandemic is reflected in the strain on mental health that reportedly began around the same time as the COVID-19 outbreak and city closure; the correlation between both can be further investigated.

Towards the end of the telehealth period, outside referrals from community health centers, such as those working with South American asylum seekers, were also accepted. ​ With time, organic appointments began coming in through the newly established phone line while the screening calls started showing diminishing returns. It was also increasingly apparent that the clinic needed to be open in person to increase the number of patients seen, as the number of appointments per week declined with time. With chronic musculoskeletal pain and diabetes / hypertension follow-ups being the most common chief complaints for telehealth appointments, it became apparent that patients suffering chronic conditions were most in need of clinic services during the COVID-19 pandemic. Many patients had gone months without medication refills and lab results they were expecting from onset of clinic closure. The COVID-19 pandemic further exacerbated existing gaps in these patients’ medical care.

While our clinic is well equipped to handle acute or urgent conditions, as a student-run free clinic which has a constant rotation of many volunteering providers representing different fields of healthcare, our clinic has had difficulty addressing the needs of chronically ill patients even before the pandemic. Such patients would benefit from seeing a consistent primary-care provider who has experience in treating and managing conditions such as diabetes and hypertension. This weakness was magnified by the clinic's virtual format. Many patients went without their medications or monitoring of their conditions starting from the city shutdown. Many others were lost to follow-up. In hopes of addressing these needs of our patients, since the reopening of the clinic, a grant was secured to host patient training programs for chronic conditions at the clinic. Through this program, patients will be provided the nutritional training and equipment needed to monitor their conditions more effectively. An experimental systematic follow-up program has also been implemented for more consistent longitudinal care for patients enrolled.

## 5 Conclusions

The San Antonio Refugee Health Clinic responded to the COVID-19 pandemic, and subsequent closure, by constructing a multi-level, systematic method of contacting patients in order to assess the needs of the local refugee community while delivering ongoing quality care. Through this initiative, patients connected with providers via telehealth calls, received medication refills and blood work, and were referred to psychiatric, social, and dental resources as requested along with proper follow-up. Limited access to WiFi and webcam devices proved to be a major limitation to providing healthcare in the virtual setting. Our data suggests that throughout the COVID-19 pandemic, the San Antonio Refugee population faced barriers to healthcare accessibility, job security, access to social services, and mental health. Of note, our study does not discern whether the pandemic is a direct cause of any of these challenges as it does not address how the patients’ burdens changed before and after the onset of the pandemic. Nevertheless, we hope that our refugee-centered community outreach adds to the discourse surrounding how to best provide equitable services for vulnerable populations in times of need.

## Declaration of Competing Interest

The authors declare that they have no known competing financial interests or personal relationships that could have appeared to influence the work reported in this paper.
